# Association of Child Mental Health with Child and Family Characteristics in Rural China: A Cross-Sectional Analysis

**DOI:** 10.3390/ijerph18105107

**Published:** 2021-05-12

**Authors:** Huan Wang, Cody Abbey, Xinshu She, Scott Rozelle, Xiaochen Ma

**Affiliations:** 1 Stanford Center on China’s Economy and Institutions, Stanford University, Stanford, CA 94305, USA; huanw@stanford.edu (H.W.); cjabbey@stanford.edu (C.A.); rozelle@stanford.edu (S.R.); 2 Stanford School of Medicine, Stanford University, Stanford, CA 94305, USA; xinshe@stanford.edu; 3 China Center for Health Development Studies, Peking University, Beijing 100871, China

**Keywords:** mental health, rural school children, China, strengths and difficulties questionnaire

## Abstract

Assessing the mental health problems encountered by school children and understanding the contributing factors are crucial to inform strategies aimed at improving mental health in low-resource contexts. However, few studies have investigated the mental health problems among disadvantaged children in poorer countries. This study examines the prevalence of mental health problems in rural China and their association with child and family characteristics. The study uses survey data from 9696 children in 120 rural primary schools and measures child mental health using the Strengths and Difficulties Questionnaire (SDQ). Overall, 17.9% of the sample children were found to be in the abnormal range of the SDQ total difficulties scores. The mean score was 12.93 (SD = 4.94). Abnormal scores were associated with child and family characteristics, including older child age (Odds Ratio, OR = 0.704, 95% CI: 0.611, 0.810; *p* < 0.001), gender (OR = 1.235, 95% CI: 1.112, 1.371; *p* < 0.001), and academic performance (OR = 0.421, 95% CI: 0.369, 0.480; *p* < 0.001). Reading time was found to be protective for mental health. Risk factors include excessive screen time (OR = 1.685, 95% CI: 1.409, 2.016; *p* < 0.001) and being bullied (OR = 3.695, 95% CI: 3.301, 4.136; *p* < 0.001). Our study suggests that future mental health illness prevention programs in rural China should consider targeting different aspects of children’s social contexts.

## 1. Introduction

Mental health problems are significant contributors to the disease burden of children across the globe [1]. Globally, it is estimated that 13.4% of children aged 6–18 are at risk of mental health problems such as anxiety or depression [2], and mental health problems account for 15–30% of the disability-adjusted life years lost during the first three decades of life [3]. Mental illnesses, if left untreated, can result in significant economic burdens on society [4]. Childhood is a critical period for mental health interventions, as during this developmental stage there is a rapid increase in the prevalence of mental health disorders, and episodes during this period increase the risk of illness later on in adulthood [5].

Accurate estimates of the prevalence of child mental illness in developing countries, as well as the identification of potential risk and protective factors, are essential for setting up adequate and timely services and diminishing the long-term economic as well as health consequences of child mental disorders. Compared to developed countries, larger shares of the population in developing countries tend to live in remote rural areas, where there is often a vast gap between mental health needs and available resources [6]. The combination of increased exposure to risk factors such as poverty and the paucity of local screening and treatment services make rural children particularly vulnerable to poor mental health outcomes [7].

A substantial share of the child population in LMICs live in China, where there appears to be a high prevalence of mental health problems among children in general, but there is a relative lack of evidence related to the mental health of rural children. China’s school children population of approximately 270 million is one of the largest in the world, making child mental health in China to be of particular importance globally. Previous studies reported the prevalence of mental health illnesses among China’s school children to be between 10% and 57% [8,9,10]. However, most of these studies focused solely on children living in urban areas, which is problematic since more than half of the 0–19 year-old population live in rural areas [11]. Moreover, the current healthcare system in China has a severe shortage of pediatricians who specialize in child psychiatry, and in rural areas of inland provinces, well-trained specialists are particularly scarce [12,13]. Thus, more attention to the mental health of rural children is necessary for further understanding the state of child mental health in China overall and demonstrating the urgency of widening mental healthcare access to vulnerable children in remote areas.

The current literature on the mental health of rural children in China has a number of gaps, including the examination of a relatively limited number of risk factors and small sample sizes. One of the few large-scale urban-rural comparisons of child mental health in China which used the Strengths and Difficulties Questionnaire (SDQ) discovered that children living in rural communities had less prosocial behavior and had more emotional symptoms, behavioral problems, and peer interaction problems than their urban peers, though this study did not examine risk factors besides student gender, age, and regional economic level [14]. A number of more recent studies found similar or worse outcomes for rural children using the same screening tool, though these were more limited in terms of geographic scope and sample size [15,16].

Children in rural China may face a higher risk of having mental health problems for several reasons. First, there is a high prevalence of “left-behind” children whose parents migrate to urban areas for better job opportunities [17]. Studies found evidence suggesting that left-behind children’s mental health, relative to that of their rural peers, is poor [18,19], which may be due to the lack of a core support system at home [20]. Second, a high proportion of rural children live in school-supplied boarding facilities [21]. Research demonstrates a negative association between school boarding and mental health [22,23], as boarding children also lack the social support of their parents [24]. Third, a recent literature documented high rates of bullying in China, particularly in rural areas [25,26]. Bully victims are especially vulnerable to mental health issues [27]. Fourth, rural students in China perform significantly worse than their urban peers in school [28,29,30,31,32]. Feelings of academic incompetence or failure to succeed were shown to increase internalizing symptoms among children [33,34]. Finally, the absence of parental monitoring may lead to a high prevalence of excessive screen time among rural children [35,36], which may have adverse implications for their mental health.

Assessing mental health problems among rural children and understanding the potential contributing factors may inform strategies aimed at improving their mental health development. However, the mental health problems of children in rural China are understudied. To fill these gaps, we first measure the prevalence of mental health problems among the 9696 children in our rural sample. Second, we investigate the associations between mental health problems and background characteristics on the individual child level (age, gender, boarding status, and academic performance) and family level (parental migration, education, and family assets). Moreover, we explore the associations between mental health problems and child behavioral risk and protective factors (screen time, reading time, and experience being bullied in school).

## 2. Materials and Methods

This study uses cross-sectional data collected in June 2018 in three rural counties located in one prefecture of Jiangxi, which is a province located in southeastern China. These three sample counties were randomly selected from the sample prefecture. The economic development in the three counties is close to the average of counties in rural China as well as of other areas in the Jiangxi province. Per capita yearly income in the three counties was 1255 USD in 2015, which is close to the national median income (1737 USD) of rural residents [37,38]. In addition, more than 80% of the population are rural residents [39]. These three sample counties are thus roughly representative of rural counties in general, where nearly half of China’s population lives.

### 2.1. Ethical Approval

Ethical approval for this study was granted by the Stanford University Institutional Review Board (IRB) (Protocol ID 32594). We adhered to the Declaration of Helsinki throughout the survey in terms of both maintaining privacy and ensuring confidentiality. To maintain privacy, students filled out the forms individually and no discussion of the data was allowed during the survey at each school. To ensure confidentiality, immediately after data processing, the paper-based survey forms (with identifiable information) were placed into a locked filing cabinet accessible only to the head of field research. Student names were then deleted from all electronic files, with only a survey code linking them together with the original paper-based forms. All electronic data were stored in an encrypted computer.

### 2.2. Participants and Procedure

To select our sample, we followed a two-step sample selection protocol. The first step involved selecting a representative sample of schools from the three counties. As this study was supported by the local education authorities, our local research team was able to obtain official records from county education bureaus to create a population frame of all rural, public primary schools in the three counties. According to the records, there was a total of 458 schools. We then randomly selected 120 schools to be included in our sample. Of these, 37 schools (30.8%) were in County A; 25 schools (20.8%) were in County B; and 58 schools (48.3%) were in County C. In this way, our sample is representative of the three counties being studied.

The second step was to sample classes. We conducted our study among fifth and sixth grades in each of the sample schools. Due to financial constraints, we randomly selected at most two classes in each grade in each school. Specifically, if there were only one or two classes in a grade, all classes in this grade were selected. If there were more than two classes in a grade, we randomly selected two classes.

In June 2018, a team of enumerators composed of 120 undergraduate and graduate students at a local university carried out the data collection in each sample classroom. Prior to the survey, all enumerators had attended a two-day training session. Written consent forms detailing the purpose and content of the survey were also sent to parents or guardians of eligible children in the sample schools. On the day of the survey in each classroom, enumerators introduced the survey content and protocol to all sample students and obtained their oral assent, adhering to official guidelines regarding human subject research ethics of studies involving children [40,41]. All 10,112 children in the sample classes who were present on the day of the survey (100%) gave their oral assent to participate and were included. Ultimately, a total of 9696 (95.8%) of these children returned survey forms with complete data and were included in analysis.

The survey included questionnaires printed on paper which were filled out by each child and collected data on student mental health, basic demographic information, after-school time allocation, and bullying victimization. Students also participated in a standardized math test. The enumerators adhered to a strict protocol for each part of the survey and enforced strict time limits for the standardized test.

### 2.3. Measures

#### 2.3.1. Mental Health Measures

Mental health problems were measured by a self-report version of the Strengths and Difficulties Questionnaire (SDQ). The SDQ is a well-recognized psychiatric screening instrument for children. It was adapted and validated for use in China, demonstrating strong internal consistency (Cronbach’s alpha coefficient = 0.81) and high levels of reliability (Pearson’s correlation coefficient = 0.71) [42,43]. The questionnaire includes 25 items, each of which is scored on a three-point scale (0 = not true, 1 = somewhat true, and 2 = certainly true). The SDQ is divided between the following five sub-scales, each of which has five items in total: emotional problems, conduct problems, hyperactivity and inattention, peer problems, and prosocial behavior. The highest possible score on each sub-scale is 10 points, with higher scores on the first four scales and a lower score on the fifth scale indicating poorer mental health outcomes. The sum of the first four scales generates a total difficulties score ranging from 0 to 40. The prosocial scale, which measures strengths, is not included in the total score [44]. The score on each individual sub-scale, as well as the total score, places children into one of three categories according to the cutoffs validated for Chinese children: normal, borderline, or abnormal [45]. Children whose scores are classified as “abnormal” are at greater risk of having mental problems compared with their peers. The abnormal score ranges for the scale and each sub-scale are as follows: total difficulties score: 17–40, emotional problems score: 5–10, conduct problems score: 4–10, hyperactivity score: 7–10, peer problems score: 4–10, and prosocial score: 0–4.

#### 2.3.2. Demographic Information

All child demographic and behavioral information was collected using self-report, Chinese-language questionnaires that students filled out during the survey. This information included age (years) as well as gender (1 = boy, 0 = girl). We later categorized student age into the following categories: 8–11 years old, 12 years old, and 13–15 years. Information on whether children were boarding (1 = yes, 0 = no) was also collected. We also asked whether both parents migrated out for work for more than six months in the past year (1 = yes, 0 = no) and then classified those children whose parents were both migrants as “left-behind children”.

In the questionnaire, the students also responded to a number of questions related to household information. To report the education level of their parents, their answers indicated the highest level of education their parents had (primary school, junior high, high school, or college and above). To measure socioeconomic status, the questionnaire also asked whether or not their household owned seven selected items included in the National Household Income and Expenditure Survey (1 = yes, 0 = no) [46]. From this information, we generated an index of family assets, and families were categorized into bottom, middle, and top terciles.

#### 2.3.3. Academic Performance

To measure academic performance, the survey team administered a standardized, 30-min math test to students, which included test items developed by local education experts that are appropriate for children in the fifth and sixth grades. Test scores were then categorized into bottom, middle, and top terciles.

#### 2.3.4. After-School Time Allocation

To measure the daily after-school time allocation of the sample children, children were asked to fill out on an average school day how many minutes they spend engaging in (a) extracurricular reading and (b) activities involving screen time on either a smartphone or a computer (such as internet browsing or video games). Responses were categorized into none, less than half an hour, half an hour to one hour, one hour to two hours, and more than two hours.

#### 2.3.5. Bullying Victimization

Information on bullying victimization was collected using a widely used “Students Bullied at School” (SBS) scale. The SBS scale was developed for the Progress in Reading and Literacy Study (PIRLS), which is the largest international project that assesses academic achievement among children across 52 countries and regions, representing a variety of development and income levels. The scale has been validated in China [47]. On the eight-question scale, students are asked, “During this school year, how often have other students from your school done any of the following things to you (including through texting or the Internet)?” Possible responses include “never”, “a few times a year”, “once or twice a month”, or “at least once a week”. Following previous studies [48], responses were categorized into three groups by frequency of bullying victimization: “almost never”, “about monthly”, and “about weekly”.

### 2.4. Statistical Analysis

We first report the summary statistics of the sample, including student and family characteristics, potential protective factors, and potential risk factors. Next, in the descriptive analysis, we reported the distribution of mental health problems measured by the Strengths and Difficulties Questionnaire. The means of the total difficulties score and sub-scale scores, as well as the proportions of children with normal, borderline, and abnormal scores, were reported.

Univariate logistic regression models were created to assess the pairwise relationships between children with mental health problems and the sample child and family characteristics. The response variable in the logistic regression models was a bivariate variable of mental health defined by scores in the abnormal range as measured by the SDQ total difficulties score (1 = abnormal, 0 = other). The exposure variables include potential protective factors and potential risk factors, and the potential confounding covariates include student and family characteristics. Bivariate variables were used for testing the differences in mental health between each subgroup.

To adjust for confounders, a multivariate logistic regression model was also performed to examine further the associations between mental health problems as well as potential risk and protective factors (extracurricular reading time, screen time, and being bullied about weekly). Confounding variables used in the univariate regression models were adjusted in the multivariate regression model, including child characteristics (age, gender, boarding status, left-behind child status, and standardized math test score) and family characteristics (father education level, mother education level, and family asset index). We selected these confounding variables based on those included in empirical studies on similar topics in past literatures [49,50].

In addition, to check the robustness of the results of the logistic regressions, analysis was conducted using the univariate and multivariate ordinary least square (OLS) regression models. The response variable in the OLS models was a continuous variable of the SDQ total difficulties score. The exposure variables include potential protective factors and potential risk factors. The covariates include the child and family characteristics mentioned above.

Significance was established at the 10% level. All analyses were conducted in Stata 14.1 (StataCorp LP, College Station, TX, USA).

## 3. Results

### 3.1. Summary Statistics of the Sample

Table 1 describes the individual and household characteristics of participating children. Most of the 9696 participants were 12 years old (45.4%)—approximately one-third were between 13 and 15 years old (30.7%), and the remaining share (23.9%) were between 8 and 11 years old. The sample was roughly balanced by gender (49.4% were female). The large majority of children boarded (89.5%). Slightly over half of the sample were left-behind children (50.4%). Almost half of fathers did not attend junior high school (44.3%), while close to two-thirds of mothers did not (63.0%).

In terms of daily extracurricular reading time, 7.1% of children reported that they did not engage in any extracurricular reading, while 28.0% reported reading for less than half an hour, 31.8% reported reading for half an hour to one hour, 25.8% reported reading for one to two hours, and 7.3% read for two or more hours. In terms of daily screen time, over two thirds (35.1%) reported no screen time, while 26.2% reported less than half an hour of screen time, 16.5% reported half an hour to one hour of screen time, 13.2% reported an hour to two hours of screen time, and 9.1% reported over two hours of screen time. Around 20.6% of the children reported being bullied weekly.

### 3.2. The Prevalence of Abnormal SDQ Scores by Subgroup

Table 2 reports the prevalence of mental health problems measured by scores in the abnormal range on the SDQ total difficulties scale and individual sub-scales. The mean total difficulties score was 12.93 (SD = 4.94). A total of 17.9% of the sample children were found to be in the abnormal range, indicating that they might have mental health problems. In terms of difficulty sub-scales, emotional symptoms (16.8%) and conduct problems (14.9%) were most prevalent. Additionally, peer problems were prevalent among 9.8% of the sample, and hyperactivity or attention problems were prevalent among 6.3% of the sample. In terms of strengths, the share of students with abnormal prosocial behavior scores (17.2%) was similar to the share with abnormal total difficulties scores.

### 3.3. Logistic Regression of Factors Associated with Abnormal Total Difficulties Scores

According to Table 3, certain characteristics were significantly associated with mental health problems. For example, a lower share of older children had total difficulties scores in the abnormal range in both the univariate logistic regression model (Odds Ratio, OR = 0.704, *p* < 0.001) and multivariate model (OR = 0.701, *p* < 0.001). Being male was associated with abnormal total difficulties scores in the univariate model (OR = 1.235, *p* < 0.001), but not in the multivariate analysis. Boarding at school and being a left-behind child were not associated with abnormal total difficulties scores. Children with higher academic performance were less likely to have abnormal total difficulties scores in both the univariate model (OR = 0.421, *p* < 0.001) and multivariate model (OR = 0.516, *p* < 0.001). Children whose parents completed junior high school were also less likely to have abnormal total difficulties scores compared to children whose parents did not complete junior high school, but the association was not significant after adjusting for confounding factors. Family asset level was also not associated with abnormal total difficulties scores on the SDQ.

In both the univariate and multivariate logistic models, we found that extracurricular reading time was a protective factor for student mental health. There was a clear trend that as children’s daily extracurricular reading time increased, the likelihood of having an abnormal total difficulties score decreased. For example, compared to children who did not engage in daily extracurricular reading, children who read for half an hour to one hour per day had a significantly lower likelihood of having abnormal total difficulties scores in both the univariate model (OR = 0.751, *p* = 0.005) and multivariate model (OR = 0.751, *p* < 0.001). Children who read for more than two hours a day had an even lower likelihood of having abnormal total difficulties scores according to both the univariate model (OR = 0.567, *p* < 0.001) and multivariate model (OR = 0.671, *p* = 0.008).

Risk behavioral factors for poor mental health included self-reported screen time of two or more hours per day (OR = 1.685, *p* < 0.001) and being bullied at school (OR = 3.695, *p* < 0.001). In addition, we observed a dose-dependent effect of the association between abnormal total difficulties scores and screen time. Compared to those with no screen time at all, screen time of half an hour to one hour per day was associated with a higher likelihood of having mental health problems (OR = 1.16, *p* = 0.033), while for screen time exceeding more than two hours per day the association was even stronger (OR = 1.685, *p* < 0.001).

### 3.4. OLS Regression of Factors Associated with the Total Difficulties Score

Similar results were found in the OLS analysis, in which the outcome measure of the total difficulties score of the SDQ was treated as a continuous variable (as opposed to being a limited dependent variable as in the previous three paragraphs—Table 4). Those who were older, male, and had higher test scores were still significantly more likely to have lower total difficulties scores. However, no significant association was found between total difficulties scores and boarding status, being a left-behind child, parental education level, or family assets.

In the OLS regression model, extracurricular reading time was once again found to be a protective factor and was associated with a higher level of mental health in a predominately dose-dependent fashion. In the univariate model, when compared to the reference group who did not engage in any extracurricular reading, engaging in half an hour of reading was associated with a decrease by of 0.91 points (*p* < 0.001) of the SDQ total difficulties score, while engaging in more than two hours of reading was associate with a decrease of 1.9 points (*p* < 0.001) of the SDQ total difficulties score. This result was consistent after adjusting for confounding factors in the multivariate model.

Similarly, screen time and being bullied were negatively associated with student mental health. Compared to children who did not report any screen time, worse mental health was observed for those with more screen time in a dose-dependent fashion. The total difficulties score was significantly higher for children whose screen time was half an hour to one hour (by 0.389 points, *p* = 0.003) and even higher for those whose screen time exceeded two hours (by 1.267 points, *p* < 0.001). These associations were significant even after adjusting for confounding factors in the multivariate model. In addition, being bullied at school was significantly associated with lower levels of mental health. The total difficulties score was higher for children who were bullied in school according to both the univariate model (by 3.614 points *p* < 0.001) and multivariate model (by 3.393 points, *p* < 0.001).

## 4. Discussion

A total of 17.9% of the sample children were found to be in the abnormal range of the SDQ total difficulties scale, indicating that they may be at risk of having mental health problems. According to the multivariate regression results, screen time of over 2 h per day and exposure to bullying were risk factors of higher SDQ total difficulties scores, while engaging in daily extracurricular reading was a protective factor. We also found that a number of other characteristics were associated with higher SDQ total difficulties scores, including age, being female, having a parent with a lower education level, and worse academic performance. We did not find a significant difference between the prevalence of mental health problems among left-behind children and non-left-behind children or between boarding and non-boarding students.

Overall, the mental health outcomes of the rural children in the current study were poor when compared to their peers in other studies that also use the SDQ. The average SDQ total difficulties score in our study (12.93) is higher than those reported by studies conducted in developed countries [51,52,53,54] and similar to those reported by studies conducted in rural areas of other developing countries [55,56]. Compared to total difficulties scores reported in other studies of children in China, the mean score in our study is also among the highest [42,57]. The strengths score measured by the pro-social subscale in our sample (6.41) is similar to the results of previous studies in China [58] and lower than those reported by studies in both developed and developing contexts outside of China [59,60,61].

A total of 17.9% of the sample children were found to be in the abnormal range of the total difficulties scale, indicating that they may be at risk of having mental health problems. Extrapolating this figure to all 95 million school children in rural China [62], we estimate that there are about 20 million rural children who might be at risk of having mental health problems.

Our finding that there was no significant difference between the prevalence of mental health problems among left-behind children and non-left-behind children contradicts the majority of existing studies conducted in rural China, which generally show left-behind children to be a disadvantaged subgroup in terms of psychological well-being [15,63,64,65], though there is another cross-sectional study that also found no differences [57] and a longitudinal study that found null impacts of parental migration on child mental health [32]. Mixed results on the effects of parental migration have also been identified in contexts outside of China. While left-behind children in India, Peru, and Vietnam were found to have worse health outcomes than their peers, there was no significant difference between LBC and non-LBCs in Ethiopia and the Philippines [66,67]. The non-significance possibly reflects a trade-off between an increase in household income from parents migrating to cities for better job opportunities and a decline in parental care [63]. Another potential reason is that children who grow up without their parents may learn to adapt to such adverse circumstances, thereby creating more opportunities for them to exercise agency and independence, increasing their resilience and ultimately reducing the negative impact on their mental health. This hypothesis is supported by studies conducted both in rural China and in other contexts [68,69]. It also aligns with the challenge model of resilience theory, which posits that exposure to certain levels of adversity increases one’s ability to cope with future setbacks [70]. The most direct implication is that nations where no significant differences between LBC and non-LBC outcomes exist may need universal, rather than targeted, approaches to mental health among children in poor, rural areas. Future studies could explore the reasons behind the differential impacts of parental migration in different contexts.

This study also contributes to two strands of international literature on behavioral factors related to child mental health. The first strand documents the association between sedentary behaviors and poor mental health outcomes [71,72]. Although a majority of the existing studies focused on screen time, few included non-screen sedentary behavior (e.g., extracurricular reading) [73,74,75]. Our study supports and extends upon the findings of one prior study that discovered a negative association between physical health and screen time, while finding no such association with non-screen sedentary activities [76]. The second strand of literature is the relationship between worse mental health outcomes and school bullying [25,77,78]. A review of existing studies found that there is an inadequacy in the understanding of the association between bully victimization and mental health [79]. Few past studies identified associations of bullying with mental health problems among rural Chinese children [26]. Our finding that school bully victims are 3.6 times more likely to have poor mental health as measured by the SDQ provides evidence that bullying victimization is indeed a key risk factor for poor mental health and deserves more attention in future research and school policy guidelines, especially when considering the high prevalence of weekly bullying in our sample.

Finally, our finding that a number of sociodemographic characteristics were associated with higher SDQ total difficulties scores indicates worse mental health among these subgroups. The negative association between total difficulties score and age is in line with one previous study [80] and contrasts with those that identify no clear association [60]. The higher scores among girls in our sample aligns with the findings of some studies while contrasting with others that identify no significant gender differences [26,49] or higher scores among boys [60]. Indeed, the association of factors such as age and gender with mental health may depend on the kind of disorder: Previous studies reported fewer externalizing problems (measured by the conduct and hyperactivity problem sub-scales) and more internalizing problems (measured by the emotional and peer problem sub-scales) among older children and girls [81,82]. The negative association between math test scores and total difficulties scores in our sample may indicate that academic stress is one factor causing poorer mental outcomes for low-performing students [83]. Although we cannot determine causality, it is possible that it is a bidirectional relationship [84]. Additionally, our finding that having parents with a higher educational level is a protective factor for mental health is supported by prior research [57], as parent educational attainment is associated with higher-quality parent-child interactions [85] and a more supportive home environment, which are predictive of optimal child mental health [86].

Our study has a number of strengths that allow us to contribute to both the global literature and the China-specific literature on public health. First, we identify the association between mental health and a wide range of potential risk and protective factors, some of which were rarely examined in the prior literature on child mental health, such as extracurricular reading. This has important implications for designing in-school and at-home intervention strategies aimed at improving the mental health of disadvantaged children in developing contexts. Second, because we use the SDQ—a widely used instrument that has been validated in many different countries around the world—we are able to position the mental health of rural children in China in the context of global mental health and allows for our study design to be reproduced in other contexts. Third, our study has a large sample (*n =* almost 10,000), which provides us with a large amount of statistical power to support our study’s conclusions. Fourth, our data were collected from a representative cross-section of China’s rural children, including multiple individual and family characteristics, allowing us to identify the most vulnerable subgroups in rural China.

Our study also has several limitations. First, the cross-sectional design of our study limited our ability to draw any causal inferences about the potential risk and protective factors in our results, underscoring the need for researchers to use longitudinal randomized studies to investigate the causal effects of psychosocial factors examined in this study. Second, we used a self-report screening tool, which can only estimate risk in populations. Despite the demonstrated high validity and reliability of some mental health screening tools such as the SDQ, clinical diagnoses are necessary to make formal assessments of illness and rule out other possible causes [87]. Third, although our sample is among the largest of existing studies conducted in rural China on this topic, we collected the data from schools in China’s relatively poor rural areas, which limits our ability to extrapolate our findings to China’s nonpoor areas or to areas outside of China.

## 5. Conclusions

Using data from 120 rural primary schools in China, our findings indicate that children in rural China potentially bear one of the highest risks for mental health problems in the world. Specifically, 17.9% of the sample children were found to be in the abnormal range of the SDQ total difficulties scores, and a similar share (17.2%) of children had scores in the abnormal range of the prosocial sub-scale. Our study also identifies certain psychosocial risk factors (including weekly bullying and screen time of at least two hours per day) and protective factors (30 min or more extracurricular reading per day) of child mental health. Our results add to the growing literature on the prevalence of mental health issues and correlates among rural, low-income children of LMICs, highlighting the need for examining the roles that reducing school bullying, decreasing screen time, and increasing extracurricular reading time may play in mental health illness prevention and treatment. Additionally, the association of mental health with factors at both the school level (such as bullying, which may reflect school environment rather than just individual social regulation issues) and the household level (such as parental education level, which may be a surrogate for parental social support) further suggests that future interventions should also involve the people (teachers, parents) and settings (home, school) in children’s lives [7].

## Figures and Tables

**Table 1:** Summary of child and family characteristics.

Variable	Characteristic Categories	*n* (%)
**Child characteristics**		
Age (Years)	Age 8–11	2309 (23.9%)
	Age 12	4392 (45.4%)
	Age 13–15	2969 (30.7%)
Male	No	4769 (49.4%)
	Yes	4888 (50.6%)
Boarding	No	8659 (89.5%)
	Yes	1019 (10.5%)
Left-behind child	No	4809 (49.6%)
	Yes	4887 (50.4%)
Standardized math test score	Top tercile	3224 (33.4%)
	Middle tercile	3305 (34.2%)
	Bottom tercile	3136 (32.4%)
**Family characteristics**		
Father education	Less than junior high	4153 (44.3%)
	Junior high	3940 (42.1%)
	High school	985 (10.5%)
	High school above	288 (3.1%)
Mother education	Less than junior high	5872 (63.0%)
	Junior high	2537 (27.2%)
	High school	700 (7.5%)
	High school above	207 (2.2%)
Family asset index	Top tercile	3530 (37.0%)
	Middle tercile	3316 (34.7%)
	Bottom tercile	2704 (28.3%)
**Protective and risk factors**		
Extracurricular reading time	None	683 (7.1%)
	Less than half an hour/day	2707 (28.0%)
	0.5 to 1 h/day	3083 (31.8%)
	1 to 2 h/day	2503 (25.8%)
	≥2 h/day	708 (7.3%)
Screen time	None	3395 (35.1%)
	Less than half an hour/day	2535 (26.2%)
	0.5 to 1 h/day	1599 (16.5%)
	1 to 2 h/day	1277 (13.2%)
	≥2 h/day	880 (9.1%)
Being bullied about weekly	No	7702 (79.4%)
	Yes	1994 (20.6%)

**Table 2:** The distribution of Strengths and Difficulties Questionnaire mean scores and the prevalence of abnormal, borderline, and normal scores as per established cut-offs.

Scales	Mean Score	Standard Deviation	Abnormal, *n* (%)	Borderline, *n* (%)	Normal, *n* (%)
Total SDQ difficulties score (range 0–40)	12.93	4.94	1736 (17.9%)	1803 (18.6%)	6157 (63.5%)
**By SDQ subscales, each subscale score ranges from 0 to 10**					
Emotional symptoms	3.33	2.18	1629 (16.8%)	1125 (11.6%)	6942 (71.6%)
Conduct problems	2.68	1.72	1445 (14.9%)	1348 (13.9%)	6913 (71.3%)
Hyperactivity problems	3.61	1.89	611 (6.3%)	873 (9.0%)	8213 (84.7%)
Peer problems	3.32	1.61	980 (9.8%)	1076 (11.1%)	7670 (79.1%)
Prosocial behaviors	6.41	2.15	1668 (17.2%)	1726 (17.8%)	6302 (65.0%)

**Table 3:** Logistic regression of factors associated with total difficulties scores in the abnormal score of the Strengths and Difficulties Questionnaire.

		Univariate Logistic Regression Models			Multivariate Logistic Regression Model		
Variable	Characteristics	Odds Ratio	95% CI	*p* Value	Odds Ratio	95% CI	*p* Value
**Child characteristics**							
Age (Years)	Age 8–11 (reference)						
	Age 12	0.796	(0.701,0.904)	<0.001	0.828	(0.720,0.952)	0.008
	Age 13–15	0.704	(0.611,0.810)	<0.001	0.701	(0.600,0.819)	<0.001
Male	No (reference)						
	Yes	1.235	(1.112,1.371)	<0.001	1.028	(0.912,1.159)	0.653
Boarding	No (reference)						
	Yes	1.093	(0.926,1.290)	0.293	1.011	(0.840,1.217)	0.909
Left-behind child	No (reference)						
	Yes	0.903	(0.813,1.001)	0.053	0.949	(0.846,1.066)	0.379
Standardized math test score	Bottom tercile (reference)						
	Middle tercile	0.517	(0.457,0.586)	<0.001	0.596	(0.521,0.683)	<0.001
	Top tercile	0.421	(0.369,0.480)	<0.001	0.516	(0.447,0.596)	<0.001
**Family characteristics**							
Father education	Less than junior high (reference)						
	Junior high	0.783	(0.698,0.878)	<0.001	0.946	(0.830,1.078)	0.404
	High school	0.843	(0.701,1.013)	0.068	0.877	(0.711,1.083)	0.223
	High school above	1.118	(0.834,1.498)	0.457	1.005	(0.712,1.420)	0.977
Mother education	Less than junior high (reference)						
	Junior high	0.784	(0.691,0.890)	<0.001	0.852	(0.738,0.983)	0.028
	High school	1.08	(0.886,1.315)	0.447	1.153	(0.918,1.449)	0.219
	High school above	1.4	(1.012,1.938)	0.042	1.085	(0.731,1.609)	0.686
Family asset index	Top tercile (reference)						
	Middle tercile	1.075	(0.949,1.217)	0.256	1.072	(0.935,1.229)	0.317
	Bottom tercile	1.018	(0.892,1.162)	0.788	1.056	(0.912,1.224)	0.465
**Protective factors**							
Extracurricular reading time	None (reference)						
	Less than half an hour/day	0.751	(0.616,0.917)	0.005	0.751	(0.602,0.938)	0.011
	0.5 to 1 h/day	0.672	(0.551,0.819)	<0.001	0.723	(0.579,0.902)	0.004
	1 to 2 h/day	0.559	(0.455,0.687)	<0.001	0.644	(0.512,0.811)	<0.001
	≥2 h/day	0.567	(0.433,0.741)	<0.001	0.671	(0.499,0.902)	0.008
**Risk factors**							
Screen time	None (reference)						
	Less than half an hour/day	1.16	(1.012,1.330)	0.033	1.131	(0.973,1.314)	0.110
	0.5 to 1 h/day	1.13	(0.964,1.323)	0.131	1.077	(0.903,1.285)	0.410
	1 to 2 h/day	1.196	(1.010,1.416)	0.037	1.089	(0.900,1.318)	0.381
	≥2 h/day	1.685	(1.408,2.016)	<0.001	1.717	(1.401,2.104)	<0.001
Being bullied	No (reference)						
	Yes	3.695	(3.301,4.136)	<0.001	3.655	(3.240,4.123)	<0.001

Note: Confounding variables used in univariate regression models were adjusted in the multivariate regression model, including child and family characteristics, potential protective factors, and potential risk factors.

**Table 4:** OLS regression of factors associated with the Strengths and Difficulties Questionnaire total difficulties score.

		Univariate OLS Model			Multivariate OLS Model		
Variable	Characteristics	Coefficient	95% CI	*p* Value	Coefficient	95% CI	*p* Value
**Child characteristics**							
Age (Years)	Age 8–11 (reference)						
	Age 12	−0.353	(−0.601,−0.104)	0.005	−0.200	(−0.441,0.040)	0.102
	Age 13–15	−0.562	(−0.830,−0.294)	<0.001	−0.434	(−0.695,−0.173)	0.001
							
Male	No (reference)						
	Yes	0.277	(0.080,0.474)	0.006	−0.239	(−0.437,−0.041)	0.018
Boarding	No (reference)						
	Yes	0.468	(0.147,0.788)	0.004	0.240	(−0.073,0.553)	0.133
Left-behind child	No (reference)						
	Yes	−0.239	(−0.435,−0.042)	0.017	−0.113	(−0.305,0.078)	0.247
Standardized math test score	Bottom tercile (reference)						
	Middle tercile	−1.85	(−2.083,−1.616)	<0.001	−1.428	(−1.661,−1.194)	<0.001
	Top tercile	−2.532	(−2.769,−2.295)	<0.001	−1.92	(−2.160,−1.680)	<0.001
**Family characteristics**							
Father education	Less than junior high (reference)						
	Junior high	−0.687	(−0.901,−0.472)	<0.001	−0.212	(−0.428,0.004)	0.054
	High school	−0.231	(−0.573,0.110)	0.184	−0.028	(−0.375,0.320)	0.876
	High school above	0.318	(−0.269,0.905)	0.289	0.098	(−0.510,0.706)	0.752
Mother education	Less than junior high (reference)						
	Junior high	−0.504	(−0.733,−0.274)	<0.001	−0.205	(−0.434,0.024)	0.079
	High school	0.173	(−0.213,0.560)	0.379	0.250	(−0.141,0.641)	0.210
	High school above	0.917	(0.234,1.601)	0.009	0.327	(−0.376,1.030)	0.361
Family asset index	Bottom tercile (reference)						
	Middle tercile	0.131	(−0.102,0.365)	0.27	0.084	(−0.142,0.311)	0.465
	Top tercile	0.084	(−0.163,0.330)	0.505	0.070	(−0.174,0.314)	0.572
**Protective factors**							
Extracurricular reading time	None						
	Less than half an hour/day	−0.91	(−1.322,−0.498)	<0.001	−0.806	(−1.213,−0.400)	<0.001
	0.5 to 1 h/day	−1.484	(−1.891,−1.078)	<0.001	−1.184	(−1.588,−0.781)	<0.001
	1 to 2 h/day	−1.841	(−2.256,−1.425)	<0.001	−1.409	(−1.820,−0.998)	<0.001
	≥2 h/day	−1.932	(−2.448,−1.416)	<0.001	−1.412	(−1.919,−0.905)	<0.001
**Risk factors**							
Screen time	None						
	Less than half an hour/day	0.386	(0.133,0.639)	0.003	0.281	(0.034,0.527)	0.026
	0.5 to 1 h/day	0.399	(0.106,0.692)	0.008	0.372	(0.085,0.658)	0.011
	1 to 2 h/day	0.622	(0.305,0.939)	<0.001	0.540	(0.227,0.853)	0.001
	≥2 h/day	1.267	(0.902,1.632)	<0.001	1.283	(0.920,1.647)	<0.001
Being bullied	No (reference)						
	Yes	3.614	(3.382,3.846)	<0.001	3.393	(3.157,3.629)	<0.001

Note: Confounding variables used in univariate regression models were adjusted in the multivariate regression model, including child and family characteristics, potential protective factors, and potential risk factors.

## Data Availability

Data available upon reasonable request.
